# Erie County Alms-House

**Published:** 1847-11

**Authors:** 


*Erie County Alms-House.*—From the report of the Superintendents of the poor, of the County of Erie, for the year ending Oct. 1st., we learn that the whole number relieved at that institution during the year, is 978; and the number relieved out of the institution in the city of Buffalo, 3434, making, in all, over five thousand persons. Of those relieved at the Alms-House, 204 were native born, and 744 were foreigners.

The number of persons at the Alms-House Oct. 1, were:—lunatics, 31; idiots, 11; paupers, 101; vagrants, 19; total, 162. Of these the number of adults, was 124; of children 138. The number of deaths at the Alms-House during the year was 113.

From January 1st to October 1st, 117 cases of *ship fever* were treated at the institution. We are informed by the attending Physicians, Drs. Sprague and Loomis, that about the same number were treated out of the institution, in the city, supported from the poor fund.

The Superintendents report in favor of an additional building. It is imperatively called for. In fact the limited accommodations of that institution (which years ago we endeavored to bring to the attention of the Supervisors and citizens, and which have remained nearly the same, nothwithstanding the great increase of pauperism;) are truly disgraceful to the County. Drs. G. F. Pratt, and F. L. Harris. have been appointed Physicians and Surgeons to the institution for the ensuing year, at a salary of $400.

